# Recapitulating essential pathophysiological characteristics in lung-on-a-chip for disease studies

**DOI:** 10.3389/fimmu.2023.1093460

**Published:** 2023-02-28

**Authors:** Yanning Zhang, Xuejiao Wang, Yaoqing Yang, Jing Yan, Yanlu Xiong, Wenchen Wang, Jie Lei, Tao Jiang

**Affiliations:** ^1^ Department of Thoracic Surgery, The Second Affiliated Hospital, Air Force Medical University, Xi’an, China; ^2^ State Key Laboratory for Manufacturing Systems Engineering, Xi’an Jiaotong University, Xi’an, China

**Keywords:** lung-on-a-chip, lung diseases, diagnosis, microengineering technology, pathophysiology

## Abstract

Lung diseases have become a significant challenge to public healthcare worldwide, which stresses the necessity of developing effective biological models for pathophysiological and pharmacological studies of the human respiratory system. In recent years, lung-on-a-chip has been extensively developed as a potentially revolutionary respiratory model paradigm with high efficiency and improved accuracy, bridging the gap between cell culture and preclinical trials. The advantages of lung-on-a-chip technology derive from its capabilities in establishing 3D multicellular architectures and dynamic microphysiological environments. A critical issue in its development is utilizing such capabilities to recapitulate the essential components of the human respiratory system for effectively restoring physiological functions and illustrating disease progress. Here we present a review of lung-on-a-chip technology, highlighting various strategies for capturing lung physiological and pathological characteristics. The key pathophysiological characteristics of the lungs are examined, including the airways, alveoli, and alveolar septum. Accordingly, the strategies in lung-on-a-chip research to capture the essential components and functions of lungs are analyzed. Recent studies of pneumonia, lung cancer, asthma, chronic obstructive pulmonary disease, and pulmonary fibrosis based on lung-on-a-chip are surveyed. Finally, cross-disciplinary approaches are proposed to foster the future development of lung-on-a-chip technology.

## 1 Introduction

The lungs are involved in many essential functions, including respiration, pulmonary circulation, and immunity. They are susceptible to viruses, bacteria, and other microorganisms from the external environment and inside the human body. Lung diseases can lead to respiratory failure and life-threatening conditions. Acute lung injury, as caused by the severe acute respiratory syndrome coronavirus 2 (SARS-CoV-2), poses an enormous global threat to humanity. SARS-CoV-2 mainly attacks the lungs, causing cytokine storm, acute respiratory distress syndrome, and septic shock, and can cause death in severe cases (1–3). Chronic lung injury, as caused by chronic obstructive pulmonary disease (COPD), also decreases life expectancy quality (4, 5). The prevalence of COPD is high, affecting 328 million people worldwide, especially in low- and middle-income regions (6–8). Malignant tumors, such as lung cancer, are the leading cause of death in many countries. Treating these diseases necessitates a deep understanding of the underlying mechanisms, pathological processes, and potential therapeutic targets, all of which rely on reliable physiological and pathological models.

**Figure 1:**
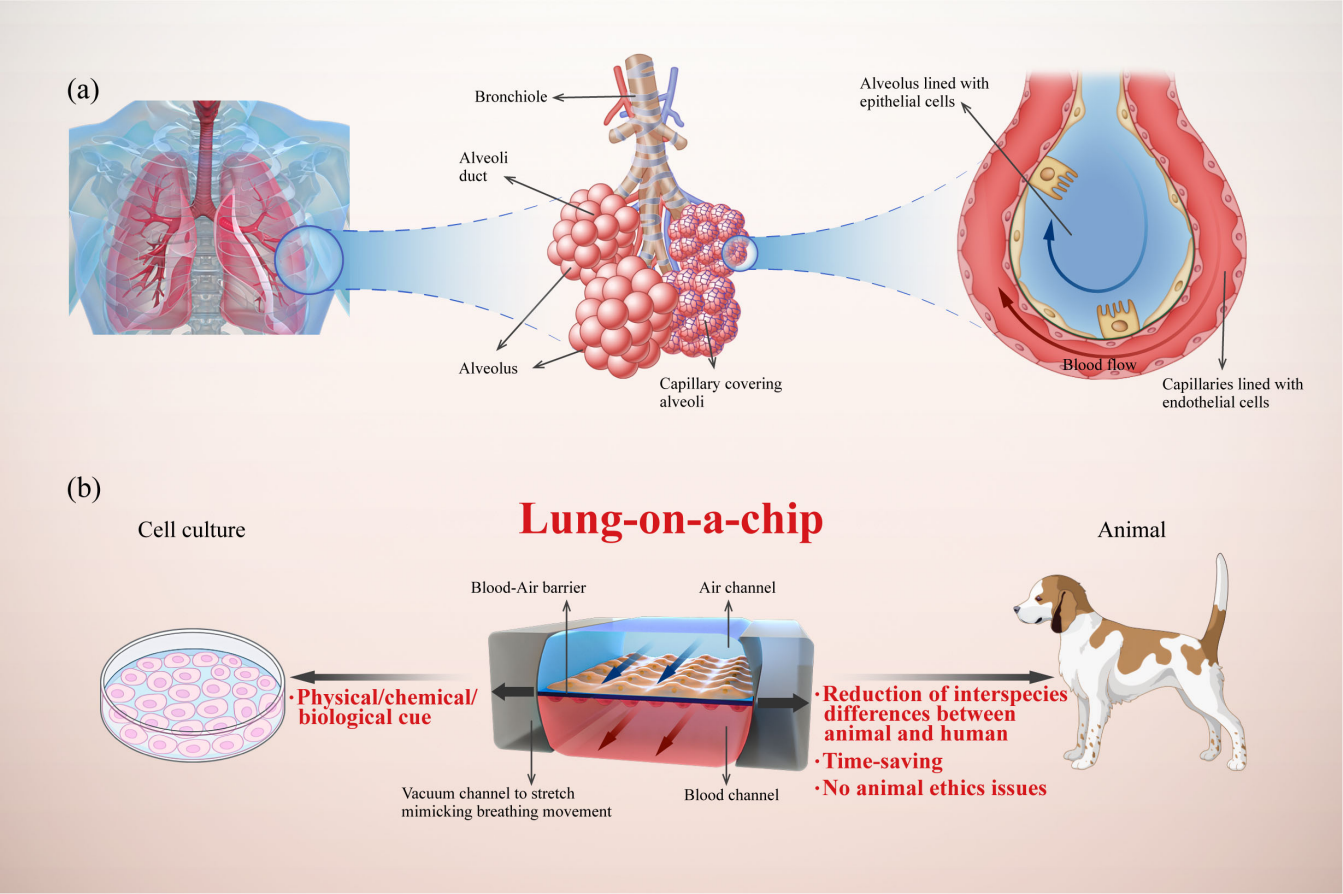
Illustration of a human lung-on-a-chip design illustrating key physiological characteristics of the alveoli and the advantages of lung-on-a-chip compared with traditional models. **(A)** Analysis of lung alveoli as functional units composed of epithelial cells, endothelial cells, and interstitium; **(B)** Typical structure of lung-on-a-chip with breathing-induced mechanical activity, and its advantages compared with cell culture and animal models.

While simple and intuitive, traditional models of 2D cell culture cannot reflect *in vivo* cell niches, as these models commonly lack the extracellular matrix (ECM), physical/chemical/biological cues, multicellular interactions, and intracellular signaling pathways (9). Furthermore, it is difficult to model the complexity of organs and communication between cells (10). As an alternative approach, animal models struggle to overcome their deviations from humans in terms of pathophysiological processes and drug responses. Specifically, there are significant structural differences between animal and human respiratory systems; in animal models, various inhalation stimuli are normally deposited on the turbinate and the upper respiratory tract and do not reach the lungs, making it difficult to recreate human pathology. In addition, the use of animal models is time-consuming and may be ethically problematic (11, 12). Clinical trials are subject to various constraints resulting from interspecific differences, ethical issues, and economic costs (**Figure 1**). All these limitations result in a bottleneck for experimental research and drug development, encouraging researchers to develop efficient and reliable platforms for preclinical drug testing.

Lung-on-a-chip combines microfluidics and cell biology to build three-dimensional (3D) structures that mimic alveoli and airways (**Figure 1**). Alveoli/airway-on-a-chip devices recapitulate alveolar epithelial, microvascular endothelial, interstitial fibroblast, and multicellular interactions. These devices can form the air–liquid interface between the epithelium and endothelium with air and blood fluid dynamics. In addition, these devices can integrate respiratory movements (13, 14), immune cells transported by microvascular perfusion (15) and the transfer between microflora (16) and other related organs (17, 18), all of which constitute powerful tools for understanding the mechanism and progress of lung diseases (19). In this review, we propose an innovative classification of core components in the lung-on-a-chip based on the pathophysiological structures and functions of the lungs. Furthermore, we summarize the latest advances in lung-on-a-chip-based research for lung inflammation, lung cancer, asthma, COPD, and pulmonary fibrosis.

## 2 Physiological structure of lungs

### 2.1 Lung conducting airways

The airway is the path to the lungs during respiration, composed of the trachea and bronchus, with 23 branches extending from the trachea to the terminal sacs. As the lumen of each branch of the alveolar duct becomes thinner, the epithelium gradually changes from pseudolaminated to single-layer ciliated columnar epithelium, where the numbers of goblet cells, glands, and cartilage decrease, and the amount of smooth muscle increases. Diastolic smooth muscle contraction can change the diameter of the airway, regulating the volume of air in the alveoli. The airways communicate with the external environment, and airway epithelial cells serve as the first line of defense against particles, pathogens, and toxins (25, 26). Smooth muscle spasms and lumen narrowing cause dyspnea in some pathological conditions, such as asthma and COPD.

### 2.2 Alveoli

Alveoli are the basic functional units of the lungs and the main component of air exchange. In adults, there are approximately 700 million bilateral alveoli, accounting for 90% of the whole lung (27); each one is a physiological unit for gas exchange between air–blood interface, composed of alveolar type I and II cells. Alveolar type I cells, covering approximately 95% of the entire alveolar surface, contribute to gas exchange with the associated endothelium. Whereas, alveolar type II cells are characterized by secretory organelles participating in innate immune responses and producing surfactants to maintain surface tension. Individual alveoli maintain good elasticity, with volumes increasing by approximately 15% during respiration. Adjacent alveoli are interdependent and interconnected through small pores. When one alveolus collapses, the tension of the surrounding alveolar walls increases, limiting further alveolar collapse and increasing stability *via* interdependence. The respiratory membrane, also known as the air–blood barrier, is important in maintaining the basic structure and microenvironment of the lungs (28, 29). The main cell components of the respiratory membrane are type I and type II alveolar epithelial cells, endothelial cells, and fibroblasts (29, 30). Lung diseases that thicken the respiratory membrane or increase the diffusion distance, such as atelectasis, emphysema, lobectomy, and capillary closure and obstruction, can slow the rate and amount of gas diffusion, reduce the respiratory membrane diffusion area, and affect pulmonary ventilation.

### 2.3 Alveolar interstitium

The alveolar interstitium is the space between the alveolar epithelium and capillary endothelial basal layer, including a variety of cells and ECM networks, such as elastic and bundled collagen fibers (31), which impart compliance and elasticity to the lungs, respectively, affecting the amount of air inhaled and the ease with which the lung retracts to its resting position after inflation (32, 33). Elastic fibers exhibit a linear stress-strain relationship over a large range facilitating lung parenchyma elastic retraction and stabilization (34, 35).

## 3 Lung-on-a-chip model construction

The lung-on-a-chip can be classified into four parts based on structural characteristics: the respiratory membrane structure, alveolar cell arrangement, breathing movement, and air–liquid interface. The typical lung-on-a-chip models are summarized in **Table 1**.

**Table 1:** Literature review of typical systems for lung-on-a-chip.

Models	Number of compartment	Cell types	ECM materials	Physiological read-outs	Advantages	Ref.
Intracellular kinetics of viral infection and the antiviral responses against it	1	Type I alveolar cells (NCI-H1703) Type II alveolar cells (NCI-H441) Lung fibroblasts (MRC5) Lung microvascular endothelial cells (HULEC-5a)	Atelocollagen	The stylus surface profiler and histological images confirmed intricate microarchitecture and morphologies as well as its major functions histological and immunohistochemical analysis validate the epithelial and endothelial cells arrangement	recapitulating the extremely thin and layered architecture and to control cell–cell and cell–extracellular matrix communication interactions by spatial arrangements of multiple cell types	(20)
The pathophysiological process of metastasis for lung cancer	3	Human bronchial epithelial cells (HBE) Human umbilical vein endothelial cells (HUVECs) Human lung fibroblast cells (HLFs) Mononuclear cells Astrocytes Osteoblasts Hepatocytes	/	Quantitative analyzed lung cancer growth, invasion and metastasis processes to the brain, bone, and liver to analyze the cell physiology and cell–cell interactions to validate the performance of metastasis	“multi-organs-on-a-chip” to explore lung cancer metastasis to the brain, bone, and liver, and to analyze the cell physiology and cell–cell interactions in a more physiologically relevant context.	(21)
Cellular alterations associated with bacterial and viral infections of the lung	2	Human primary epithelial cells (NHBE) Human umbilical vein endothelial cells (HUVECs) Monocyte-derived macrophages	/	A bacterial co-infection with bacteria and viruses induced the highest immune response regarding cytokine expression and barrier function loss	A human *in vitro* alveolus model composed of vascular and epithelial cell structures with cocultured macrophages	(22)
Lung-on-a-chip with mechanical stretch and fluidic shear stress resembling the human alveolus architecture and functions	3	Human primary alveolar epithelial Cells Lung fibroblasts Normal human lung smooth muscle cells Normal human lung fibroblasts Primary lung microvascular endothelial cells Human lung microvascular endothelial cells	Bovine type collagen	Quantitative characterization of the spatial and temporal distribution of the recruited immune cells; recapitulate *in vivo* relevant aspects of tissue functionality recreate the epithelium-stroma-endothelial interactions and control the microenvironment	a novel approach to recreate the epithelium-stroma-endothelial interactions and control the microenvironment, as required to recapitulate *in vivo* relevant aspects of tissue functionality	(23)
Human disease model of SARS-CoV-2-induced lung injury and immune responses	2	African green monkey kidney epithelial Vero E6 cells Immortalized human alveolar epithelial cells (HPAEpiC) Human lung microvasculature cell line (HULEC-5a) Human peripheral blood mononuclear cells	collagen	real-time quantitative PCR assess the potential therapeutics against SARS-CoV-2 tested the antiviral efficacy of remdesivir in the infected chip model with the addition of PBMCs in the vascular channel	recapitulate the lung injury and immune response to viral infection *in vitro* and in in real time simultaneously	(24)
human airway-on-a-chip identification of new potential treatment strategies for SARS-CoV-2	2	Primary human lung bronchial airway epithelial basal stem cells Primary human pulmonary microvascular endothelial cells	collagen type IV from human placenta	Quantitative analyzed cellular gene-expression level; immune response for infection with multiple influenza strains; inhibitory effects of FDA-approved drugs.	Providing a fast track to identify potential treatments for the current COVID-19.	(19)

### 3.1 Respiratory membrane structure

**Figure 2:**
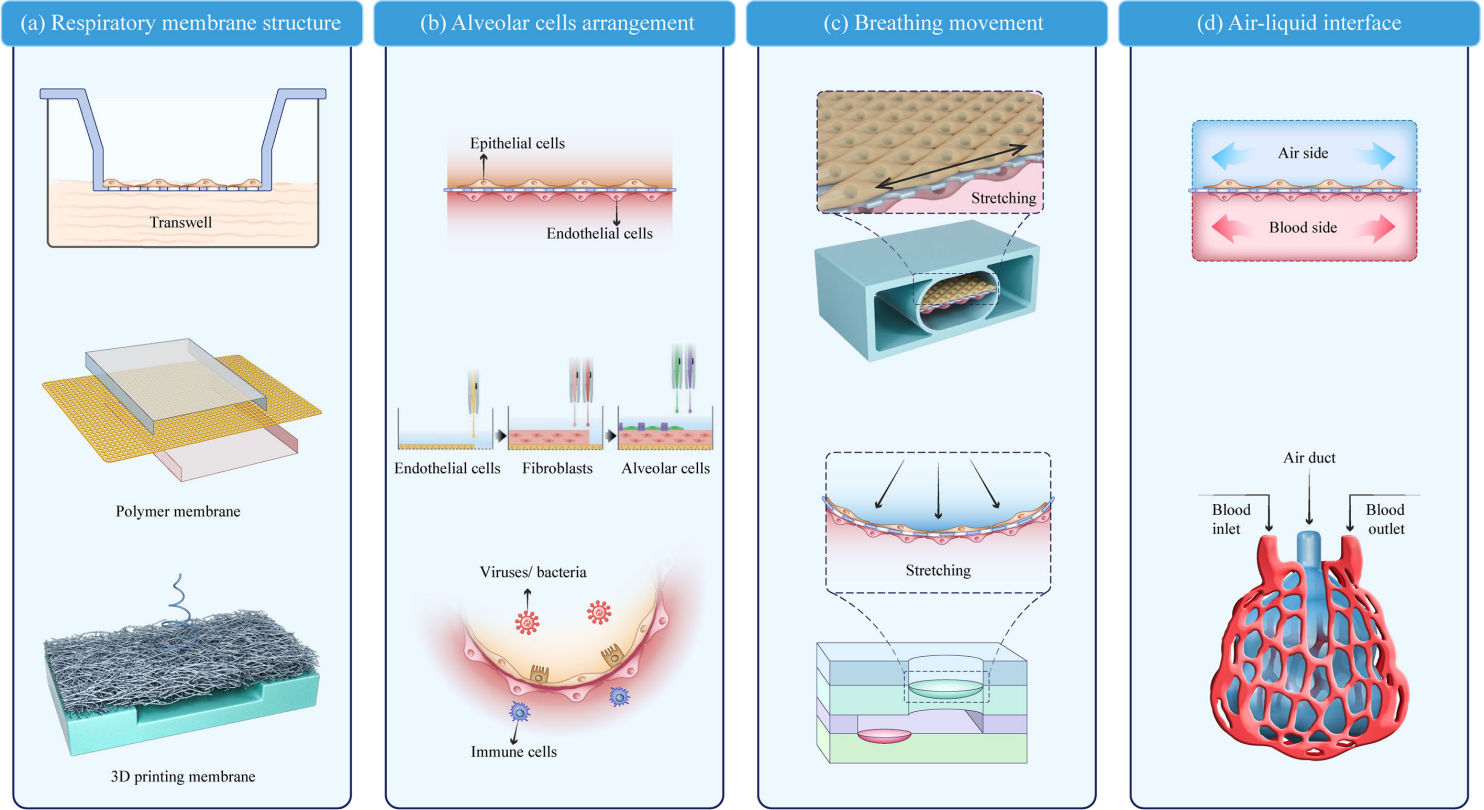
Key modules of a lung-on-a-chip. **(A)** Respiratory membrane structure; **(B)** Alveolar cell arrangement (20); **(C)** Breathing movement; **(D)** Air–liquid interface. Adapted with permission. Copyright 2021, Wiley-VCH GmbH.

As a key component of the alveolar air–liquid barrier, the membrane structure in the lung-on-a-chip has undergone three stages of development (**Figure 2A**). Early models used Transwell membranes (36). Costa A et al. (37) and Bengalli R et al. (38) grew alveolar epithelium (NCI-H441) and pulmonary microvascular endothelium (HPMUC-ST1.6R) cells on both sides of a Transwell membrane (39). The simple alveolar respiratory membrane model was used to evaluate the translocation of nanoparticles in biofilm structures and pneumonia induced by ZnO nanoparticles. However, Transwell membranes cannot be combined with microfluidic devices and lack the flexibility required for bionic design. Organosilicone-based polydimethylsiloxane (PDMS) and some thermoplastics are increasingly being used as membrane materials for lung-on-a-chips (40). PDMS is the most studied and representative membrane for ventilation and nutrient exchange (19, 39, 41). It is transparent, oxygen-permeable, stretchable, and flexible allowing the precise imitation of the alveolar dynamic mechanical deformation caused by breathing (13, 42). Other polymer films have also been widely applied and are easy to manufacture, flexible, and cost effective (43). Guan et al. (44) used polycarbonate (PC) as the membrane structure of an air–liquid interface. Air–liquid exchange membranes have also been built using polyester (PET) (45) and polymethylmethacrylate (PMMA) (9, 46). High-throughput lung organ chips with the same basic structure and environment but different cell types can integrate multi-group studies (45).

Membrane structures have some common characteristics. The pore size of the respiratory membrane is 1–10 μm, which not only ensures the exchange of nutrients and protein signal interactions of the epithelial-endothelial cells but also prevents the leakage of cells from both sides. Furthermore, to improve biocompatibility, porous polymer surfaces are typically modified with ECM-like protein materials (e.g., collagen, gelatin, and bovine fibrin). Recent fabrication techniques, such as 3D printing and bioprinting, may be valuable in achieving customized lung-on-a-chip membranes with improved biomimesis (43, 47, 48). Yang et al. (49) used PLGA nanofiber membranes as a substrate to form a uniformly sized porous network and found that different membrane thicknesses and PLGA concentrations affect the membrane permeability and ion diffusion. The printed fiber film can be adjusted to meet the design specifications and 3D-bioprinted lung models show a higher rate of proliferation and longer culture time (28 days) compared with 2D models (47).

The microporous polymer membrane acts as a barrier between the epithelial and endothelial cells, ensuring nutrient exchange and protein signal interactions between the epithelial-endothelial cells and also preventing the leakage of cells from each side. Further, the development of the lung-on-a-chip benefits from improved fabrication techniques that precisely control cell arrangement. Bioprinting technology enables the automated deposition of cells and biomaterials in 3D for highly controlled and customized production of tissue models. In constructing an alveolar model with bioinks (e.g., ECM-like hydrogel), fibroblast cells can be deposited in position with high precision. In addition, growth factors or cell inhibitors can be embedded in hydrogels to influence cell growth.

### 3.2 Alveolar cell arrangement

The arrangement of alveolar cells is a basic functional unit in the lung-on-a-chip. The typical cells found at the alveolar-capillary interface can be divided into four types: type I and II alveolar epithelial cells (A549, HPAEpiC, and NCI-H441) (43, 47, 50), microvascular endothelial cells (HUVEC, HUC-5A, HPMEC-ST1.6R, HPMEC, and Ea.hy926) (15, 41, 43, 51, 52), interstitial fibroblasts (HFL1 and MRC5) (41, 45), and other cells associated with disease models (THP-1, PMBC, and phLFs) (42, 43, 50). Endothelium/stromal cells/epithelium is the most common model arrangement, which can also involve immune cells to stimulate lung infection *in vitro* (53, 54). The interactions between the endothelial, epithelial, and immune cells provide quantitative data on unknown parameters in pulmonary infection using real-time live-cell imaging (55, 56). However, the cells used in these models grew on a semi-permeable membrane surface without a uniform arrangement, and a more biomimetic model was needed. Kang et al. (20) used inkjet printing to achieve a high-resolution arrangement of four cell types into a three-layer structure: pulmonary microvascular endothelial cells (HUC-5A) were printed on the bottom layer, pulmonary fibroblasts (MRC5) on the middle layer, and alveolar epithelial cells of type I and II on the top layer in an orderly manner, forming a respiratory membrane structure. The bionic respiratory membrane thickness was only 10 µm. To simulate the alveolar sac, DiHuang et al. (57) created an inverse opal structure using methylacrylate. The inverse opal has a vesicle and micropore connection structure highly similar to that of a human alveolar and forms a functional monolayer epithelium when filled with primary human alveolar epithelial cells. This vesicle-like structure resembles the physiological structure in the human body more closely than the previously studied planar structure (**Figure 2B**).

Cell type selection is important to the design of the lung on-a-chip. Cell lines and primary cells are both used; for example, the vascularized lung tumor-on-a-chip model consists of primary human umbilical vein endothelial cells (HUVECs) at passages 3–5, primary normal human lung fibroblasts (NHLFs) at passages 3–5, and A549 (human lung adenocarcinoma) cells (15). In a model of SARS-CoV-2 induced lung injury, the alveolar-capillary barrier is composed of human alveolar epithelial, vascular endothelial, and immune cells. In the model, the epithelial cells used are the immortalized human alveolar epithelial cell line (HPAEpiC), the vascular endothelial cells are the human lung microvasculature cell line (HULEC-5a), and the immune cells are primary isolated peripheral human blood mononuclear cells (24). Whether to use primary cells or cell lines has been extensively discussed in previous review articles (58, 59). In general, primary cells possess characteristics similar to the phenotype in the native environment, which is preferable in lung-on-a-chip models. However, it is difficult to maintain the functionality of primary cells over an extended period of culture time. Cell lines have been widely utilized due to their facile handling and growth, although they are limited by exhibiting similar functions in the original lung and airway.

Cellular organization has become more refined and closer to the native physiological structure; however, some biomimetic deficiencies still persist. For example, type I and type II alveolar epithelial cells are unlikely to stay in one alveolar space; they can pass through the alveolar interval and the epithelial tissue lining structure, and contribute to the secretion function on both sides of the alveolar interval (29). Through the use of secretory organelles, type II alveolar epithelial cells secrete surfactants that maintain the surface tension. However, to our best knowledge, this level of structural and functional complexity has not been replicated in *in vitro* alveoli models to date.

### 3.3 Breathing movement

Dynamic respiratory motion is also a key component in lung-on-a-chip construction (60). A typical breathing-mimic model consists of a 2D planar stretching surface controlled by a vacuum either side of the airway perpendicular to the membrane. Ingber et al. constructed porous membranes connecting both sides of the airflow chambers in a lung-on-a-chip using soft lithography and chemical etching. When a vacuum was applied, the constriction and deformation of the chambers induced stretching and deformation of the porous membrane attached to the epithelium/endothelial tissue. The porous film returned to its natural state when the vacuum was removed. The physiological parameters of respiration in alveoli are a cyclic strain frequency of 0.2 Hz and tensile strain of 10% (13). Compared with the Transwell static air–liquid culture, this vacuum method is a significant improvement in respiratory behavior simulation. However, plane stretching cannot completely simulate alveolar expansion, and an arc-shaped expansion movement is more bionic. Furthermore, the alveolar-like breathing motion has been gradually developed in the lung-on-a-chip, bringing the model closer to physiological pulmonary ventilation and alveoli breathing-induced stretching activity (**Figure 2C**).

### 3.4 Air–liquid interface

The air–liquid interface separating the air chamber from the blood chamber serves as the structural foundation for pulmonary gas and nutrient exchange (31, 54, 55). The air and blood channels can be integrated into a lung-on-a-chip using microfluidic channels. Models containing microfluidic channels are dynamic bionics and closer to the human respiratory membrane than the traditional semi-permeable membranes (**Figure 2D**).

In the lungs, the blood flows through the pulmonary microvascular networks. The lung-on-a-chip design must take two factors into account. First, the shear stress of circulating blood on the endothelium as it flows through the vascular system and across the cell surface. Endothelial cells are subjected to a shear stress in the range of 1–10 dyn/cm^2^ (56), which regulates cell behaviors including proliferation, differentiation, cell information interaction, and barrier formation (61). Second, the pulmonary microvascular network plays a significant role in the immune response. Neutrophils and other immune cells circulate in the blood and create vascularized immunity. The airflow channel in the lung-on-a-chip not only simulates respiration and communication with the outside environment but also contains different substances (e.g., small particles of PM 2.5 dust, viruses, and bacteria) to model the inhalation causing different lung diseases, such as asthma (62), COPD (63), influenza (64), pneumonia (25), and tuberculosis (65). Airflow and blood flow have been integrated into the same chip to reproduce both air and blood transport. Miller and Stevens (66) used projection stereolithography to develop 3D multivascular transport regimes. Oxygenation and human red blood cell flow during tidal ventilation have also been explored.

A nutrient supply is essential for the growth and functional expression of a variety of cells on the air–liquid interface. The basic medium is available during the culture of a lung-on-a-chip, and growth factors can also be used to meet the endothelial, epithelial, and immune cell needs. In particular, the patient’s serum can be used for the culture component in the blood flow channels. The lung-on-a-chip often contains different chambers and microfluidic channels; the medium can be confined to certain cell chambers and different chambers can be connected according to the cell needs.

### 3.5 Physiologic and molecular read outs

Various characterization techniques have been used to analyze the lung-on-a-chip models. Cell-cell interactions and microenvironment and physical parameters can be detected to verify the physiological conditions and pathological progress *in vivo*. Most conventional techniques in cell and tissue biology can be used as off-line bioassays for the lung-on-a-chip, including fluorescence staining, western blotting, and PCR (20, 67). For example, immunofluorescence microscopic images using specific antibodies: the endothelial marker CD31 derived from HULEC-5a cells, the tight junction protein ZO-1, and hydrophilic surfactant SP-A secreted by type II alveolar cells. The alveolar barrier model was stained with hematoxylin & eosin and SARS-CoV-2 infection was predominantly identified in the epithelium layer by viral spike protein expression. RNA-seq analysis showed distinctive immune responses to SARS-CoV-2 infection in cocultured HPAEpiC cells and HULEC-5a cells on chips (24). To analyze the biochemical changes in the microenvironment of the lung-on-a-chip, chemokines and cytokines can be collected. For example, the medium is collected to compare the levels of IL-8, IL-6, IL-1 β, MCP-1, and GM-CSF secreted by hAECs in the chips (57). Chiu et al. constructed a signal amplification sensing film to detect the cytokeratin 19 fragment (68). The lung-on-a-chip can have built-in electrodes for TEER biosensors and micro-impedance tomography. Oxygen, temperature, and lung disease biomarker sensors also measure the physical parameters of the lung-on-a-chip (69, 70). For example, an organ- and disease-specific *in vitro* mini lung fibrosis model equipped with noninvasive real-time monitoring of cell mechanics has been introduced. The real-time measurement of cell/tissue stiffness and compliance is a clinical biomarker of the progression/attenuation of fibrosis upon drug treatment, which is confirmed for inhaled Nintedanib—an antifibrosis drug (43). Gao et al. designed a giant magnetoresistance multi biomarker immunoassay that can detect 12 kinds of tumors (71, 72).

## 4 Lung disease studies based on lung-on-a-chip

### 4.1 Pneumonia

Pneumonia is an inflammation of the lung parenchyma including the terminal airways, alveolar cavities, and interstitial spaces. This inflammation can cause endothelial and epithelial cell damage, apoptosis, respiratory barrier disruption, capillary dilation, leukocyte infiltration, and massive release of inflammatory factors.

Alveoli-on-a-chip can be used as a platform for pneumonia research by introducing pro-inflammatory factors (TNF-α) or stimulants (LPS/silica/zinc oxide nanoparticles) into the vascular microchannels to stimulate the airway; most of the irritants are inhaled through airway aerosolization to simulate exposure to biological species in the environment (13, 38, 73, 74). High-concentration ultrafine particles (UFP, less than 100 nm) in the environment are likely to cause respiratory system inflammation and negatively impact health. Fine particles enter the bloodstream *via* the air–liquid barrier and interact with immune cells, affecting the vascular endothelium and other tissues. Camatini et al. (38) investigated the toxicology of zinc oxide nanoparticles (nZnO) using a Transwell model and discovered that nZnO activates alveolar epithelial and endothelial cells to release inflammatory mediators (IL-6 and IL-8). Immune system monocytes (THP-1) can modulate the epithelial response to nZnO. However, the molecular mechanism of action is not known. Kooter et al. (75) used an exposure model to investigate transcriptomic responses in the epithelium of healthy and asthmatic airways exposed to different copper oxide nanoparticle aerosols.

Viruses, bacteria, and other pathogenic microorganisms are common pathogenic factors in pneumonia; primary influenza viral infection and bacterial coinfection significantly increase mortality. After infection, alveolar damage and massive macrophage infiltration cause inflammatory activation of the lung, which cannot be replicated in traditional models. Endothelial injury caused by primary influenza virus infection and *Staphylococcus aureus* co-infection was studied in a human alveolar model (76). Co-infection leads to a significant impairment of the endothelial barrier integrity, and immune cell inflammation resulting in lung injury. Moreover, the shear stress of the blood cell and macrophage flow strengthens the barrier function. To model influenza infection in the upper respiratory cortex, Jung et al. (20) constructed an ultrathin high-resolution 3D alveolar model by inkjet printing, and influenza A H1N1 virus (PR8) was blown into the epithelial airway side of the lung-on-a-chip. Both NCI-H441 (type II epithelium) and MRC5 (lung fibroblast) cells were extremely sensitive to influenza infection.

The COVID-19 pandemic poses a serious threat to human health. Research models that replicate organ-level physiology are critical for understanding the COVID-19 pathogenesis. Studies on SARS-CoV-2 virus-host interactions using the lung-on-a-chip support the effective diagnosis and treatment of COVID-19. COVID-19-induced pulmonary microvascular injury and immune response in the lung-on-a-chip demonstrate that viral infection causes endothelial injury accompanied by alveolar barrier damage, which is more severe in the presence of PBMC (42). The model revealed a complex crosstalk among alveolar epithelial, endothelial, and host immune responses that are not readily realized in cell and animal models. The lung-on-a-chip has also been used to evaluate the feasibility of antiviral therapy against SARS-CoV-2 in preclinical studies. Remdesivir is an antiviral compound against multiple RNA viruses and has been approved by the FDA (77, 78). After three days of administering remdesivir into the airway of a lung chip model infected with SARS-CoV-2, viral replication was inhibited, resulting in significant therapeutic effects and alleviation of the barrier disruption. This platform could be used to test drug candidates (anti-inflammatory cytokine inhibitors).

### 4.2 Lung cancer

Lung cancer is the second most commonly diagnosed cancer and leading cause of cancer death in 2020, representing 11.4% of all diagnosed cancers and 18.0% of deaths. It is also the leading cause of cancer-related morbidity and mortality in men, followed by that of women (4). It is important to understand the basic biological characteristics of lung cancer, such as infinite proliferation, apoptosis resistance, and migratory movements, to develop treatment strategies. Lung-on-a-chip technology can enable tumors to grow, develop, and interact within their own microenvironment.

Yang et al. (49) established a PLGA-based lung-on-a-chip with cocultured human fetal lung fibroblasts (HFL1) and NSCLC (A549) to evaluate gefitinib sensitivity. Insulin-like growth factor (IGF-1) secreted by HFL1 cells mitigated the inhibitory effect of gefitinib on the EGFR signaling pathway by activating the PI3K/Akt signaling pathway, promoting tumor cell growth, and reducing sensitivity to gefitinib. Furthermore, it was discovered that A549 cells could cause endothelial cell apoptosis or death, followed by tumor invasion when HUVECs were introduced into this model.

Lung tumor-on-a-chip has also used isolated cells from primary lung cancers (CAFs and ECs) (15, 79) to accurately design and reproduce cell-cell communication in the lung tumor microenvironment (80). Isolated CAFs from lung adenocarcinomas were investigated for their effect on A549 cell migration (81). NSCLC cell lines in the lung tumor-on-a-chip are influenced by mechanical stretching (82) and blood fluid flow (78, 83) during lung cancer progression and drug response. The lung tumor-on-a-chip from the primary tumors of NSCLC patients under dynamic perfusion has been used to characterize tumor-immune interactions through autologous tumor-infiltrating lymphocytes (84) and can predict patient specificity for immune checkpoint blocking therapy (85). These studies investigated individual-specific tumor immunobiology and drug responses, which is a potential future study direction for lung-on-a-chip techniques (80).

Lung cancer metastasis is a complex physiological process, and lung-on-a-chip can simulate metastasis by integrating multi-organ chips. A multi-organ-on-a-chip consists of an upstream “lung” and three downstream “distal organs,” and it uses a multi-channel microfluidic chip to mimic the *in vivo* microenvironment in lung cancer metastasis. Bronchial epithelium, lung cancer cells, microvascular endothelial cells, monocytes, and fibroblasts grow on either side of the biofilm in the upstream “lung,” and astrocytes, osteocytes, and hepatocytes grow in distal compartments, mimicking lung cancer metastasis to the brain, bone, and liver. Furthermore, quantitative analysis is used to replicate lung cancer growth, invasion, and metastasis. Preclinical *in vitro* models should accommodate the interactions between tumors and immune cells, preferably including individual tumor cells harvested directly from patient biopsies as our understanding of the complexity of lung cancer development and metastasis is limited.

### 4.3 Asthma and COPD

Asthma is characterized by reactive spasms of the small airways, and COPD by chronic damage to the small airways and alveoli. The main characteristics of asthma include chronic airway inflammation, airway hyperresponsiveness to various stimuli, reversible airflow restriction, and a series of structural changes in the airway with a prolonged disease course (86). COPD is the third leading cause of death worldwide, affecting 200 million people (5, 87, 88). COPD is usually caused by the accumulation of harmful particles or gases in the airways or alveoli, and inflammatory cells such as neutrophils, macrophages, and T lymphocytes are involved in the pathogenesis. Small airways are essential for delivering air to the lungs and excreting secretions, and they are the primary sites of exposure to environmental factors; therefore, they are closely involved in such diseases as COPD and asthma (25, 89).

To establish a small airway model that closely mimics the physiological microenvironment associated with COPD, cell culture with an air–liquid interface (ALI) is used. Based on an *in vitro* COPD model, Chen et al. (36) determined the differences in the expression and characteristics of the autophagic protein LC3B between human normal and COPD small airway epithelial cells. LC3B affects the differentiation of COPD cells into basal, secretory, mucous, and ciliated cells. The spreading patterns and morphology changes of the blood vessels in the airways affect airway remodeling, resulting in irreversible airway obstruction that aggravates asthma (90, 91). To better illuminate the functions of the blood vessels in asthma, Nam et al. (52) fabricated an airway model with 3D printing technology featuring an interface between tracheal epithelium and perfusable blood vessels. This asthma disease model demonstrated that it is possible to imitate the tissue infiltration of immune cells, which is the initiation of an active immune/inflammatory response in asthma patients. Furthermore, asthma enhances the sensitivity of the airways to nanoparticle aerosols (75), possibly as a combined result of a hyperactive airway and inefficient mucociliary clearance mechanisms.

### 4.4 Lung fibrosis

Lung fibrosis is a chronic and fatal disease featuring fibroblast proliferation, abnormal ECM deposition, stiffening of lung tissue, and loss of lung function in the end (92, 93). In addition to using simple ALI models using Transwell membranes under static conditions, an increasing number of pulmonary fibrosis studies have focused on the lung-on-a-chip (13, 94–96). Felder et al. (97) developed a respirable lung chip to examine the effects of human liver growth factor (rhHGF) and physiological-cycle mechanical stretching. Cyclic mechanical stretching significantly hindered wound healing, while rhHGF could partially improve wound healing. These findings help elucidate the complex pathogenesis of lung fibrosis. Sundarakrishnan et al. fabricated 3D bioengineered pulmonary fibrotic (Eng-PF) tissues recreating the pathology of human fibroblastic foci (Hum-FF). This pulmonary fibrosis-on-a-chip incorporated different components to simulate various aspects of IPF, including epithelial injury with bleomycin and cellular recruitment by perfusion of cells through the hydrogel microchannel (98). A more recent study described a comprehensive organ/disease-specific model that recapitulated the key attributes of pulmonary fibrosis and the conditions during inhalation therapy (43). The pulmonary fibrosis chip used in the study not only imitated the microenvironment of alveolar cells but also allowed for real-time measurement of tissue stiffness or compliance, which are key parameters used for clinical diagnostics of the progression/attenuation of pulmonary fibrosis. With such results as enhanced tissue compliance and reduced collagen formation, this study demonstrated the effectiveness of aerosolized nintedanib, an FDA-approved antifibrotic drug, in the treatment of lung fibrosis.

## 5 Future perspectives

The lung-on-a-chip, a fledgling technology that mimics the human pulmonary environment, not only surpasses classical cell culture models but also reduces our reliance on animal models to elucidate the complex pathophysiology of lung diseases and accelerate drug development. However, several aspects of the technology could be further improved, and this may require multidisciplinary cooperation. First, since the lung-on-a-chip technology is based on physiological simulations, an in-depth knowledge of lung anatomy, function, and disease progression is critical for model development. The application of novel biotechnologies, such as single-cell sequencing and spatial transcriptomics, can lead to a deeper and more comprehensive understanding of the lung microenvironment, and may offer more insights for better lung-on-a-chip models. Second, emerging technologies such as 3D printing, gene editing, and high-resolution imaging can integrate biosensors into the lung-on-a-chip to monitor cell behavior, environmental parameters (oxygen content, metabolites, etc.), and pathological processes in real time, allowing for a more realistic, accurate, and timely evaluation of disease progression and interventional treatment effects. Third, the mechanical properties, chemical cues, and biomolecules of the lung microenvironment have a significant effect on lung disease progression. Therefore, appropriately developing and modifying materials is crucial for the construction of effective lung-on-a-chip models.

## Author contributions

TJ, YX, and YZ contributed to conception and design of the study. YZ, XW, and WW organized the database. YZ and YY wrote the first draft of the manuscript. JY and XW wrote sections of the manuscript. All authors contributed to the article and approved the submitted version.

## References

[1] Hui KPY Cheung MC Perera R Ng KC Bui CHT Ho JCW. Tropism, replication competence, and innate immune responses of the coronavirus sars-Cov-2 in human respiratory tract and conjunctiva: An analysis in ex-vivo and in-vitro cultures. Lancet Respir Med (2020) 8(7):687–95. doi: 10.1016/S2213-2600(20)30193-4 PMC7252187 32386571

[2] Zhu N Zhang D Wang W Li X Yang B Song J. A novel coronavirus from patients with pneumonia in China 2019. New Engl J Med (2020) 382(8):727–33. doi: 10.1056/NEJMoa2001017 31978945 PMC7092803

[3] Huang C Wang Y Li X Ren L Zhao J Hu Y. Clinical features of patients infected with 2019 novel coronavirus in wuhan, China. Lancet (2020) 395(10223):497–506. doi: 10.1016/S0140-6736(20)30183-5 31986264 PMC7159299

[4] Sung H Ferlay J Siegel RL Laversanne M Soerjomataram I Jemal A. Global cancer statistics 2020: Globocan estimates of incidence and mortality worldwide for 36 cancers in 185 countries. CA: A Cancer J Clin (2021) 71(3):209–49. doi: 10.3322/caac.21660 33538338

[5] Wang C Xu J Yang L Xu Y Zhang X Bai C. Prevalence and risk factors of chronic obstructive pulmonary disease in China (the China pulmonary health [Cph] study): A national cross-sectional study. Lancet (2018) 391(10131):1706–17. doi: 10.1016/s0140-6736(18)30841-9 29650248

[6] Chronic Obstructive Pulmonary Disease (Copd). (2022). World Health Organization. Available at: https://www.who.int/zh/news-room/fact-sheets/detail/chronic-obstructive-pulmonary-disease-(copd)2022.

[7] Eisner MD Anthonisen N Coultas D Kuenzli N Perez-Padilla R Postma D. An official American thoracic society public policy statement: Novel risk factors and the global burden of chronic obstructive pulmonary disease. Am J Respir Crit Care Med (2010) 182(5):693–718. doi: 10.1164/rccm.200811-1757ST 20802169

[8] Global Status Report on Noncommunicable Diseases. (2014).

[9] Shrestha J Razavi Bazaz S Aboulkheyr Es H Yaghobian Azari D Thierry B Ebrahimi Warkiani M. Lung-on-a-Chip: The future of respiratory disease models and pharmacological studies. Crit Rev Biotechnol (2020) 40(2):213–30. doi: 10.1080/07388551.2019.1710458 31906727

[10] Schimek K Frentzel S Luettich K Bovard D Rutschle I Boden L. Human multi-organ chip Co-culture of bronchial lung culture and liver spheroids for substance exposure studies. Sci Rep (2020) 10(1):7865. doi: 10.1038/s41598-020-64219-6 32398725 PMC7217973

[11] de Jong M Maina T. Of mice and humans: Are they the same?–implications in cancer translational research. J Nucl Med (2010) 51(4):501–4. doi: 10.2967/jnumed.109.065706 20237033

[12] Zanoni M Piccinini F Arienti C Zamagni A Santi S Polico R. 3d tumor spheroid models for in vitro therapeutic screening: A systematic approach to enhance the biological relevance of data obtained. Sci Rep (2016) 6:19103. doi: 10.1038/srep19103 26752500 PMC4707510

[13] Huh D Matthews BD Mammoto A Montoya-Zavala M Hsin HY Ingber DE. Reconstituting organ-level lung functions on a chip. Science (2010) 328(5986):1662–8. doi: 10.1126/science.1188302 PMC8335790 20576885

[14] Stucki AO Stucki JD Hall SR Felder M Mermoud Y Schmid RA. A lung-on-a-Chip array with an integrated bio-inspired respiration mechanism. Lab chip (2015) 15(5):1302–10. doi: 10.1039/c4lc01252f 25521475

[15] Paek J Park SE Lu Q Park KT Cho M Oh JM. Microphysiological engineering of self-assembled and perfusable microvascular beds for the production of vascularized three-dimensional human microtissues. ACS nano (2019) 13(7):7627–43. doi: 10.1021/acsnano.9b00686 31194909

[16] Jalili-Firoozinezhad S Gazzaniga FS Calamari EL Camacho DM Fadel CW Bein A. A complex human gut microbiome cultured in an anaerobic intestine-on-a-Chip. Nat Biomed Eng (2019) 3(7):520–31. doi: 10.1038/s41551-019-0397-0 PMC6658209 31086325

[17] Herland A Maoz BM Das D Somayaji MR Prantil-Baun R Novak R. Quantitative prediction of human pharmacokinetic responses to drugs *Via* fluidically coupled vascularized organ chips. Nat Biomed Eng (2020) 4(4):421–36. doi: 10.1038/s41551-019-0498-9 PMC8011576 31988459

[18] McAleer CW Long CJ Elbrecht D Sasserath T Bridges LR Rumsey JW. Multi-organ system for the evaluation of efficacy and off-target toxicity of anticancer therapeutics. Sci Transl Med (2019) 11(497):aav1386. doi: 10.1126/scitranslmed.aav1386 31217335

[19] Si L Bai H Rodas M Cao W Oh CY Jiang A. A human-Airway-on-a-Chip for the rapid identification of candidate antiviral therapeutics and prophylactics. Nat Biomed Eng (2021) 5(8):815–29. doi: 10.1038/s41551-021-00718-9 PMC8387338 33941899

[20] Kang D Park JA Kim W Kim S Lee HR Kim WJ. All-Inkjet-Printed 3d alveolar barrier model with physiologically relevant microarchitecture. Adv Sci (2021) 8(10):2004990. doi: 10.1002/advs.202004990 PMC8132150 34026463

[21] Xu Z Li E Guo Z Yu R Hao H Xu Y. Design and construction of a multi-organ microfluidic chip mimicking the in vivo microenvironment of lung cancer metastasis. ACS Appl mater interfaces (2016) 8(39):25840–7. doi: 10.1021/acsami.6b08746 27606718

[22] Deinhardt-Emmer S Rennert K Schicke E Cseresnyes Z Windolph M Nietzsche S. Co-Infection with staphylococcus aureus after primary influenza virus infection leads to damage of the endothelium in a human alveolus-on-a-Chip model. Biofabrication (2020) 12(2):025012. doi: 10.1088/1758-5090/ab7073 31994489

[23] Varone A Nguyen JK Leng L Barrile R Sliz J Lucchesi C. A novel organ-chip system emulates three-dimensional architecture of the human epithelia and the mechanical forces acting on it. Biomaterials (2021) 275:120957. doi: 10.1016/j.biomaterials.2021.120957 34130145

[24] Zhang M Wang P Luo R Wang Y Li Z Guo Y. Biomimetic human disease model of sars-Cov-2 induced lung injury and immune responses on organ chip system. Adv Sci (2020) 8(3):2002928. doi: 10.1002/advs.202002928 PMC7646023 33173719

[25] Gohy S Hupin C Ladjemi MZ Hox V Pilette C. Key role of the epithelium in chronic upper airways diseases. Clin Exp Allergy (2020) 50(2):135–46. doi: 10.1111/cea.13539 31746062

[26] Nicod LP. Pulmonary defence mechanisms. Respiration (1999) 66(1):2–11. doi: 10.1159/000029329 9973683

[27] Reifenrath R. The significance of alveolar geometry and surface tension in the respiratory mechanics of the lung. Respiration Physiol (1975) 24(2):115–37. doi: 10.1016/0034-5687(75)90107-3 1242239

[28] Schittny JC. Development of the lung. Cell Tissue Res (2017) 367(3):427–44. doi: 10.1007/s00441-016-2545-0 PMC5320013 28144783

[29] Knudsen L Ochs M. The micromechanics of lung alveoli: Structure and function of surfactant and tissue components. Histochem Cell Biol (2018) 150(6):661–76. doi: 10.1007/s00418-018-1747-9 PMC6267411 30390118

[30] Gehr P Bachofen M Weibel ER. The normal human lung: Ultrastructure and morphometric estimation of diffusion capacity. Respiration Physiol (1978) 32(2):121–40. doi: 10.1016/0034-5687(78)90104-4 644146

[31] Structural Organization of the Pulmonary Interstitium. (1991).

[32] Fredberg JJ Kamm RD. Stress transmission in the lung: Pathways from organ to molecule. Annu Rev Physiol (2006) 68(1):507–41. doi: 10.1146/annurev.physiol.68.072304.114110 16460282

[33] Wilson TA Bachofen H. A model for mechanical structure of the alveolar duct. J Appl Physiol (1982) 52(4):1064–70. doi: 10.1152/jappl.1982.52.4.1064 7085408

[34] Suki B Stamenović D Hubmayr R. Lung parenchymal mechanics. Compr Physiol (2011) . p:1317–51. doi: 10.1002/cphy.c100033 PMC3929318 23733644

[35] Yuan H Kononov S Cavalcante FSA Lutchen KR Ingenito EP Suki B. Effects of collagenase and elastase on the mechanical properties of lung tissue strips. J Appl Physiol (2000) 89(1):3–14. doi: 10.1152/jappl.2000.89.1.3 10904029

[36] Chen SL Chou HC Lin KC Yang JW Xie RH Chen CY. Investigation of the role of the autophagic protein Lc3b in the regulation of human airway epithelium cell differentiation in copd using a biomimetic model. Mater Today Bio (2022) 13:100182. doi: 10.1016/j.mtbio.2021.100182 PMC8668979 34917923

[37] Costa A de Souza Carvalho-Wodarz C Seabra V Sarmento B Lehr CM. Triple Co-culture of human alveolar epithelium, endothelium and macrophages for studying the interaction of nanocarriers with the air-blood barrier. Acta biomater (2019) 91:235–47. doi: 10.1016/j.actbio.2019.04.037 31004840

[38] Bengalli R Gualtieri M Capasso L Urani C Camatini M. Impact of zinc oxide nanoparticles on an in vitro model of the human air-blood barrier. Toxicol Lett (2017) 279:22–32. doi: 10.1016/j.toxlet.2017.07.877 28709982

[39] Vera D García-Díaz M Torras N Álvarez M Villa R Martinez E. Engineering tissue barrier models on hydrogel microfluidic platforms. ACS Appl Mater Interfaces (2021) 13(12):13920–33. doi: 10.1021/acsami.0c21573 33739812

[40] Sontheimer-Phelps A Hassell BA Ingber DE. Modelling cancer in microfluidic human organs-on-Chips. Nat Rev Cancer (2019) 19(2):65–81. doi: 10.1038/s41568-018-0104-6 30647431

[41] Liu W Song J Du X Zhou Y Li Y Li R. Akr1b10 (Aldo-keto reductase family 1 B10) promotes brain metastasis of lung cancer cells in a multi-organ microfluidic chip model. Acta biomater (2019) 91:195–208. doi: 10.1016/j.actbio.2019.04.053 31034948

[42] Zhang M Wang P Luo R Wang Y Li Z Guo Y. Biomimetic human disease model of sars-Cov-2-Induced lung injury and immune responses on organ chip system. Adv Sci (Weinh) (2021) 8(3):2002928. doi: 10.1002/advs.202002928 33173719 PMC7646023

[43] Doryab A Taskin MB Stahlhut P Groll J Schmid O. Real-time measurement of cell mechanics as a clinically relevant readout of an in vitro lung fibrosis model established on a bioinspired basement membrane. Adv Mater (2022) 34(41):e2205083. doi: 10.1002/adma.202205083 36030365

[44] Guan M Tang S Chang H Chen Y Chen F Mu Y. Development of alveolar-Capillary-Exchange (Ace) chip and its application for assessment of Pm2.5-induced toxicity. Ecotoxic Environ Saf (2021) 223:112601. doi: 10.1016/j.ecoenv.2021.112601 PMC8421056 34385060

[45] Mejias JC Nelson MR Liseth O Roy K. A 96-well format microvascularized human lung-on-a-Chip platform for microphysiological modeling of fibrotic diseases. Lab chip (2020) 20(19):3601–11. doi: 10.1039/d0lc00644k 32990704

[46] Cao X Ashfaq R Cheng F Maharjan S Li J Ying G. A tumor-on-a-Chip system with bioprinted blood and lymphatic vessel pair. Adv Funct mater (2019) 29(31):1807173. doi: 10.1002/adfm.201807173 33041741 PMC7546431

[47] Wang X Zhang X Dai X Wang X Li X Diao J. Tumor-like lung cancer model based on 3d bioprinting. 3 Biotech (2018) 8(12):501. doi: 10.1007/s13205-018-1519-1 PMC6258569 30498674

[48] Rahmani Dabbagh S Rezapour Sarabi M Birtek MT Mustafaoglu N Zhang YS Tasoglu S. 3d bioprinted organ-on-Chips. Aggregate (2022), e197. doi: 10.1002/agt2.197

[49] Yang X Li K Zhang X Liu C Guo B Wen W. Nanofiber membrane supported lung-on-a-Chip microdevice for anti-cancer drug testing. Lab chip (2018) 18(3):486–95. doi: 10.1039/c7lc01224a 29309077

[50] Zhang B Korolj A Lai BFL Radisic M. Advances in organ-on-a-Chip engineering. Nat Rev Mater (2018) 3(8):257–78. doi: 10.1038/s41578-018-0034-7

[51] Park JY Ryu H Lee B Ha DH Ahn M Kim S. Development of a functional airway-on-a-Chip by 3d cell printing. Biofabrication (2018) 11(1):015002. doi: 10.1088/1758-5090/aae545 30270851

[52] Nam H Choi YM Cho S Gao G Kim D Kim J. Modular assembly of bioprinted perfusable blood vessel and tracheal epithelium for studying inflammatory respiratory diseases. Biofabrication (2022) 15(1):014101. doi: 10.1088/1758-5090/ac93b6 36130590

[53] Bastacky J Lee CY Goerke J Koushafar H Yager D Kenaga L. Alveolar lining layer is thin and continuous: Low-temperature scanning electron microscopy of rat lung. J Appl Physiol (1995) 79(5):1615–28. doi: 10.1152/jappl.1995.79.5.1615 8594022

[54] Weibel ER. Morphological basis of alveolar-capillary gas exchange. Physiol Rev (1973) 53(2):419–95. doi: 10.1152/physrev.1973.53.2.419 4581654

[55] Maina JN West JB. Thin and strong! the bioengineering dilemma in the structural and functional design of the blood-gas barrier. Physiol Rev (2005) 85(3):811–44. doi: 10.1152/physrev.00022.2004 15987796

[56] Buchanan CF Verbridge SS Vlachos PP Rylander MN. Flow shear stress regulates endothelial barrier function and expression of angiogenic factors in a 3d microfluidic tumor vascular model. Cell Adhesion Migration (2014) 8(5):517–24. doi: 10.4161/19336918.2014.970001 PMC4594487 25482628

[57] Huang D Liu T Liao J Maharjan S Xie X Perez M. Reversed-engineered human alveolar lung-on-a-Chip model. Proc Natl Acad Sci U.S.A. (2021) 118(19):e2016146118. doi: 10.1073/pnas.2016146118 33941687 PMC8126776

[58] Ahadian S Civitarese R Bannerman D Mohammadi MH Lu R Wang E. Organ-on-a-Chip platforms: A convergence of advanced materials, cells, and microscale technologies. Adv healthcare mater (2018) 7(2):1700506. doi: 10.1002/adhm.201800734 29034591

[59] Yesil-Celiktas O Hassan S Miri AK Maharjan S Al-kharboosh R Quiñones-Hinojosa A. Mimicking human pathophysiology in organ-on-Chip devices. Adv Biosyst (2018) 2(10):1800109. doi: 10.1002/adbi.201800109

[60] Douville NJ Zamankhan P Tung YC Li R Vaughan BL Tai CF. Combination of fluid and solid mechanical stresses contribute to cell death and detachment in a microfluidic alveolar model. Lab Chip (2011) 11(4):609–19. doi: 10.1039/c0lc00251h 21152526

[61] Krüger-Genge A Blocki A Franke R-P Jung F. Vascular endothelial cell biology: An update. Int J Mol Sci (2019) 20(18):4411. doi: 10.3390/ijms20184411 31500313 PMC6769656

[62] Papi A Brightling C Pedersen SE Reddel HK. Asthma. Lancet (2018) 391(10122):783–800. doi: 10.1016/s0140-6736(17)33311-1 29273246

[63] López-Campos JL Tan W Soriano JB. Global burden of copd. Respirology (2016) 21(1):14–23. doi: 10.1111/resp.12660 26494423

[64] Krammer F Smith GJD Fouchier RAM Peiris M Kedzierska K Doherty PC. Influenza. Nat Rev Dis Primers (2018) 4(1):3. doi: 10.1038/s41572-018-0002-y 29955068 PMC7097467

[65] Mandell LA Niederman MS. Aspiration pneumonia. New Engl J Med (2019) 380(7):651–63. doi: 10.1056/NEJMra1714562 30763196

[66] Grigoryan B Paulsen SJ Corbett DC Sazer DW Fortin CL Zaita AJ. Multivascular networks and functional intravascular topologies within biocompatible hydrogels. Science (2019) 364(6439):458–64. doi: 10.1126/science.aav9750 PMC7769170 31048486

[67] Jung O Tung Y-T Sim E Chen Y-C Lee E Ferrer M. Development of human-derived, three-dimensional respiratory epithelial tissue constructs with perfusable microvasculature on a high-throughput microfluidics screening platform. Biofabrication (2022) 14(2):025012. doi: 10.1088/1758-5090/ac32a5 PMC10053540 35166694

[68] Cheng S Hideshima S Kuroiwa S Nakanishi T Osaka T. Label-free detection of tumor markers using field effect transistor (Fet)-based biosensors for lung cancer diagnosis. Sensors Actuators B: Chem (2015) 212:329–34. doi: 10.1016/j.snb.2015.02.038

[69] Maoz BM Herland A Henry OYF Leineweber WD Yadid M Doyle J. Organs-on-Chips with combined multi-electrode array and transepithelial electrical resistance measurement capabilities. Lab chip (2017) 17(13):2294–302. doi: 10.1039/c7lc00412e 28608907

[70] Henry OY Villenave R Cronce MJ Leineweber WD Benz MA Ingber DE. Organs-on-Chips with integrated electrodes for trans-epithelial electrical resistance (Teer) measurements of human epithelial barrier function. Lab chip (2017) 17(13):2264–71. doi: 10.1039/C7LC00155J PMC5526048 28598479

[71] Ding S Zhang H Wang X. Microfluidic-Chip-Integrated biosensors for lung disease models. Biosensors (2021) 11(11):456. doi: 10.3390/bios11110456 34821672 PMC8615803

[72] Gao Y Huo W Zhang L Lian J Tao W Song C. Multiplex measurement of twelve tumor markers using a gmr multi-biomarker immunoassay biosensor. Biosensors Bioelectron (2019) 123:204–10. doi: 10.1016/j.bios.2018.08.060 30174274

[73] Huh D Leslie DC Matthews BD Fraser JP Jurek S Hamilton GA. A human disease model of drug toxicity–induced pulmonary edema in a lung-on-a-Chip microdevice. Sci Trans Med (2012) 4(159):159ra47–ra47. doi: 10.1126/scitranslmed.3004249 PMC8265389 23136042

[74] Huh D Leslie DC Matthews BD Fraser JP Jurek S Hamilton GA. A human disease model of drug toxicity-induced pulmonary edema in a lung-on-a-Chip microdevice. Sci Trans Med (2012) 4(159):159ra47. doi: 10.1126/scitranslmed.3004249 PMC8265389 23136042

[75] Kooter I Ilves M Grollers-Mulderij M Duistermaat E Tromp PC Kuper F. Molecular signature of asthma-enhanced sensitivity to cuo nanoparticle aerosols from 3d cell model. ACS Nano (2019) 13(6):6932–46. doi: 10.1021/acsnano.9b01823 PMC6750904 31188557

[76] Tarbell JM. Shear stress and the endothelial transport barrier. Cardiovasc Res (2010) 87(2):320–30. doi: 10.1093/cvr/cvq146 PMC2915475 20543206

[77] Wang M Cao R Zhang L Yang X Liu J Xu M. Remdesivir and chloroquine effectively inhibit the recently emerged novel coronavirus, (2019-ncov) in vitro. Cell Res (2020) 30(3):269–71. doi: 10.1038/s41422-020-0282-0 PMC7054408 32020029

[78] Yin W Mao C Luan X Shen DD Shen Q Su H. Structural basis for inhibition of the rna-dependent rna polymerase from sars-Cov-2 by remdesivir. Science (2020) 368(6498):1499–504. doi: 10.1126/science.abc1560 PMC7199908 32358203

[79] Lee SW Kwak HS Kang MH Park YY Jeong GS. Fibroblast-associated tumour microenvironment induces vascular structure-networked tumouroid. Sci Rep (2018) 8(1):2365. doi: 10.1038/s41598-018-20886-0 29403007 PMC5799156

[80] Del Piccolo N Shirure VS Bi Y Goedegebuure SP Gholami S Hughes CCW. Tumor-on-Chip modeling of organ-specific cancer and metastasis. Adv Drug Delivery Rev (2021) 175:113798. doi: 10.1016/j.addr.2021.05.008 34015419

[81] Li Z Guo T Fang L Li N Wang X Wang P. Macc1 overexpression in Carcinoma−Associated fibroblasts induces the invasion of lung adenocarcinoma cells *Via* paracrine signaling. Int J Oncol (2019) 54(4):1367–75. doi: 10.3892/ijo.2019.4702 30720137

[82] Hassell BA Goyal G Lee E Sontheimer-Phelps A Levy O Chen CS. Human organ chip models recapitulate orthotopic lung cancer growth, therapeutic responses, and tumor dormancy in vitro. Cell Rep (2017) 21(2):508–16. doi: 10.1016/j.celrep.2017.09.043 29020635

[83] Kalinowska D Grabowska-Jadach I Liwinska M Drozd M Pietrzak M Dybko A. Studies on effectiveness of ptt on 3d tumor model under microfluidic conditions using aptamer-modified nanoshells. Biosensors Bioelectron (2019) 126:214–21. doi: 10.1016/j.bios.2018.10.069 30423478

[84] Moore N Doty D Zielstorff M Kariv I Moy LY Gimbel A. A multiplexed microfluidic system for evaluation of dynamics of immune-tumor interactions. Lab Chip (2018) 18(13):1844–58. doi: 10.1039/c8lc00256h 29796561

[85] Beckwith AL Velásquez-García LF Borenstein JT. Microfluidic model for evaluation of immune checkpoint inhibitors in human tumors. Adv Healthcare Mater (2019) 8(11):1900289. doi: 10.1002/adhm.201900289 31056856

[86] Burgstaller G Oehrle B Gerckens M White ES Schiller HB Eickelberg O. The instructive extracellular matrix of the lung: Basic composition and alterations in chronic lung disease. Eur Respir J (2017) 50(1):1601805. doi: 10.1183/13993003.01805-2016 28679607

[87] Ferkol T Schraufnagel D. The global burden of respiratory disease. Ann Am Thorac Soc (2014) 11(3):404–6. doi: 10.1513/AnnalsATS.201311-405PS 24673696

[88] Quaderi SA Hurst JR. The unmet global burden of copd. Global health Epidemiol Genomics (2018) 3:e4. doi: 10.1017/gheg.2018.1 PMC5921960 29868229

[89] Brand-Saberi BEM Schäfer T. Trachea: Anatomy and physiology. Thorac Surg Clinics (2014) 24(1):1–5. doi: 10.1016/j.thorsurg.2013.09.004 24295654

[90] Zanini A Chetta A Imperatori AS Spanevello A Olivieri D. The role of the bronchial microvasculature in the airway remodelling in asthma and copd. Respir Res (2010) 11:132. doi: 10.1186/1465-9921-11-132 20920222 PMC2955663

[91] Pascual RM Peters SP. Airway remodeling contributes to the progressive loss of lung function in asthma: An overview. J Allergy Clin Immunol (2005) 116(3):477–86. doi: 10.1016/j.jaci.2005.07.011 16159612

[92] Hardie WD Glasser SW Hagood JS. Emerging concepts in the pathogenesis of lung fibrosis. Am J Pathol (2009) 175(1):3–16. doi: 10.2353/ajpath.2009.081170 19497999 PMC2708789

[93] Richeldi L Collard HR Jones MG. Idiopathic pulmonary fibrosis. Lancet (2017) 389(10082):1941–52. doi: 10.1016/s0140-6736(17)30866-8 28365056

[94] Wollin L Maillet I Quesniaux V Holweg A Ryffel B. Antifibrotic and anti-inflammatory activity of the tyrosine kinase inhibitor nintedanib in experimental models of lung fibrosis. J Pharmacol Exp Ther (2014) 349(2):209–20. doi: 10.1124/jpet.113.208223 24556663

[95] Lehmann M Buhl L Alsafadi HN Klee S Hermann S Mutze K. Differential effects of nintedanib and pirfenidone on lung alveolar epithelial cell function in ex vivo murine and human lung tissue cultures of pulmonary fibrosis. Respir Res (2018) 19(1):175. doi: 10.1186/s12931-018-0876-y 30219058 PMC6138909

[96] Felder M Stucki AO Stucki JD Geiser T Guenat OT. The potential of microfluidic lung epithelial wounding: Towards in vivo-like alveolar microinjuries. Integr Biol quantit Biosci nano to macro (2014) 6(12):1132–40. doi: 10.1039/c4ib00149d 25205504

[97] Felder M Trueeb B Stucki AO Borcard S Stucki JD Schnyder B. Impaired wound healing of alveolar lung epithelial cells in a breathing lung-on-a-Chip. Front bioeng Biotechnol (2019) 7:3. doi: 10.3389/fbioe.2019.00003 30746362 PMC6360510

[98] Sundarakrishnan A Zukas H Coburn J Bertini BT Liu Z Georgakoudi I. Bioengineered in vitro tissue model of fibroblast activation for modeling pulmonary fibrosis. ACS Biomater Sci Eng (2019) 5(5):2417–29. doi: 10.1021/acsbiomaterials.8b01262 33405750

